# Robust identification of regulatory variants (eQTLs) using a differential expression framework developed for RNA-sequencing

**DOI:** 10.1186/s40104-023-00861-0

**Published:** 2023-05-05

**Authors:** Mackenzie A. Marrella, Fernando H. Biase

**Affiliations:** School of Animal Sciences, Virginia Polytechnic Institute and State University, Blacksburg, VA USA

**Keywords:** Differential gene expression, eQTL analysis, Gene expression, RNA-sequencing, Single nucleotide polymorphism

## Abstract

**Background:**

A gap currently exists between genetic variants and the underlying cell and tissue biology of a trait, and expression quantitative trait loci (eQTL) studies provide important information to help close that gap. However, two concerns that arise with eQTL analyses using RNA-sequencing data are normalization of data across samples and the data not following a normal distribution. Multiple pipelines have been suggested to address this. For instance, the most recent analysis of the human and farm Genotype-Tissue Expression (GTEx) project proposes using trimmed means of M-values (TMM) to normalize the data followed by an inverse normal transformation.

**Results:**

In this study, we reasoned that eQTL analysis could be carried out using the same framework used for differential gene expression (DGE), which uses a negative binomial model, a statistical test feasible for count data. Using the GTEx framework, we identified 35 significant eQTLs (*P* < 5 × 10^–8^) following the ANOVA model and 39 significant eQTLs (*P* < 5 × 10^–8^) following the additive model. Using a differential gene expression framework, we identified 930 and six significant eQTLs (*P* < 5 × 10^–8^) following an analytical framework equivalent to the ANOVA and additive model, respectively. When we compared the two approaches, there was no overlap of significant eQTLs between the two frameworks. Because we defined specific contrasts, we identified trans eQTLs that more closely resembled what we expect from genetic variants showing complete dominance between alleles. Yet, these were not identified by the GTEx framework.

**Conclusions:**

Our results show that transforming RNA-sequencing data to fit a normal distribution prior to eQTL analysis is not required when the DGE framework is employed. Our proposed approach detected biologically relevant variants that otherwise would not have been identified due to data transformation to fit a normal distribution.

**Supplementary Information:**

The online version contains supplementary material available at 10.1186/s40104-023-00861-0.

## Background

A large body of studies have demonstrated that genetic variations have a direct or indirect impact on the development of phenotypic variation [1–5]. Such studies advanced our understanding of the genetic architecture of complex traits. More recently, the integration of large-scale genetic studies with transcriptome data have also identified genetic variants that explain variance in transcript abundance of specific genes (reviewed in [6]). The integration of multiple omics datasets, including genotypes, is an important step toward closing the biological gap that exists between genotypes and phenotypes [7].

Recent publications from the human Genotype-Tissue Expression (GTEx) [8, 9] and the cattle GTEx [10] projects have shed light on the genetic control of gene expression in large mammals. The recent findings indicate that genomic variants have a greater impact on gene expression than previously anticipated [11]. These studies have provided valuable information which will help close the critical gap between genomic variants and phenotypic variation [12, 13], especially those associated with health in humans and livestock.

Given the importance of identifying expression quantitative trait loci (eQTL) [14] to understand cell or tissue biology, several statistical approaches have emerged to allow the coordinated analysis of genomic variants and transcript abundance (reviewed by Nica and Dermitzakis [14]). While the first eQTL studies used microarray data [15], most of the analyses carried out in recent years use RNA-sequencing data. One emerging concern is the normalization of the data across samples. To that end, several methods have been used for data normalization across samples such as the trimmed mean of M-values (TMM) [16], fragments per kilobase per million reads (FPKM) [17], and transcript per million reads (TPM) [18]. These and other methods have been evaluated, and TMM might have an advantage over other methods [19]. Another concern related to eQTL analysis is that RNA-sequencing data do not follow a normal distribution, however, all statistical approaches currently employed assume that the inputted data will follow a normal distribution. Researchers have addressed this by transforming the data using the variance stabilization [20–22], log_2_ transformation [23, 24], or the inverse normal transformation [8, 10, 25, 26].

Because the principle of eQTL analysis is to identify differences in transcript abundance between genotypes [15], we reasoned that the analysis of eQTLs using transcript abundance estimated from RNA-sequencing could be carried out using the same framework used for differential gene expression. A major benefit of using such a framework is that differences in transcript abundance are tested and estimated using a negative binomial model [20, 27, 28], which is suitable for sequence count data [29, 30]. Thus, we hypothesized that biologically meaningful eQTLs would be identified without transforming RNA-sequencing data to fit a normal distribution. Here, our objective was to identify eQTLs in cattle peripheral white blood cells (PWBCs) using RNA-sequencing data and the Bioconductor [31] package “edgeR” [27, 32], which was designed for DGE analysis using the general linear model framework.

## Methods

All bioinformatics and analytical procedures are presented in Additional file 1.

### Data processing for variant detection, and variant filtering

We analyzed RNA-sequencing data from 42 heifers (*Bos taurus*, Angus × Simmental) publicly available in the GEO database: GSE103628 [33, 34] and GSE146041 [35]. First, we trimmed sequencing adapters and retained reads with an average quality score equal to or greater than 30 using Trimmomatic (v. 0.39) [36]. Then, we used Hisat2 (v.2.2.0) [37] to align the pair-end short reads to the cattle genome [38, 39] (*Bos_taurus*, ARS-UCD1.2.99), obtained from the Ensembl database [40]. Next, we used Samtools (v.1.10) [41] to filter reads that did not map, secondary alignments, alignments from reads that failed platform/vendor quality checks, and were PCR or optical duplicates. Duplicates were removed using the function “bammarkduplicates” from biobambam2 (2.0.95) [42]. The function “SplitNCigarReads” from GATK (v.4.2.2.0) [43] was then used to separate sequences with a CIGAR string, which resulted from sequencing exon-exon boundaries. Variants were then called in our data by using the functions “bcftools mpileup” and “bcftools call” from Samtools [41].

**Fig. 1:**
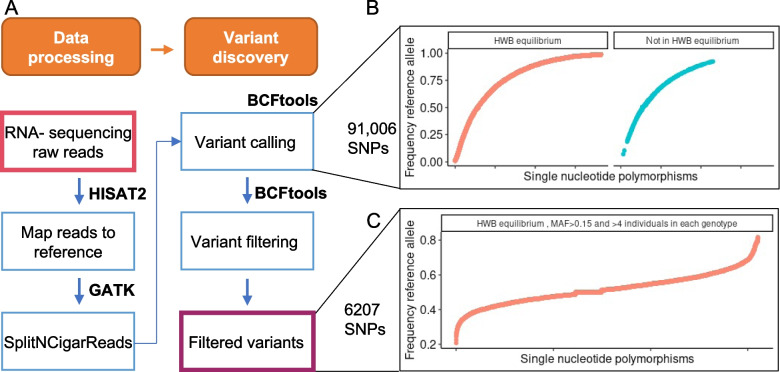
Overview of genotyping and variant discovery using RNA-sequencing data from PWBCs. **A** Schematics of bioinformatics procedures. **B** Distribution of allelic frequency of all variants genotyped in at least 26 samples. **C** Distribution of allelic frequency of all variants genotyped in at least 26 samples followed by filtering to retain 6,207 SNPs. (HW: Hardy–Weinberg; MAF: minimum allele frequency)

We filtered the variants with the function “bcftools view” from Samtools to select sites where 20 or more reads were used to identify a variant. Next, in R software (4.0.3) [44], we retained variant sites that were identified as single nucleotide polymorphisms and retained variants with genotypes called in at least 20 samples (Fig. 1A).

### Variant annotation

After the list of significant SNP-gene pairs was generated from the eQTL analysis, attributes were read in from the Ensembl genome database. The attribute list was merged with the output from the eQTL analysis as well as the nucleotide genotypic data for all samples. Ensembl Variant Effect Predictor [45] was used to compare our data to the cattle genome (*Bos taurus*, ARS-UCD1.2) to identify the functional consequences of the SNPs.

### Quantification of transcript abundance

For the expression dataset, we obtained the raw read counts from our previous work [35]. First, we eliminated one sample that had less than a million reads mapped to the annotation; second, we calculated counts per million reads (CPM) [27]; third, we retained protein-coding genes that had CPM greater than two in five or more samples. Next, we calculated TPM [46], which was used in all plots with transcript abundance.

### eQTL analysis

First, we tested whether the samples presented a genetic stratification using plink [47] to calculate the eigenvectors [48]. Given the sample elimination due to low mapping to the annotation, we carried out an eQTL analysis with 41 samples. To prevent overinflation of effects when working with variants with low allelic frequencies [49] and conduct a robust analysis with enough samples in each group of genotypes, we further retained those single nucleotide polymorphisms that had at least five animals in each of the two homozygotes and heterozygote genotypes, had a minor allelic frequency > 0.15, and followed Hardy–Weinberg equilibrium (false discovery rate = 0.05), which was tested with the R package “HardyWeinberg” [50]. In both approaches described below, eQTLs that overlapped between the ANOVA and additive model are only reported in the ANOVA model.

#### Approach 1: TMM normalized and normal-transformed RNA-seq data

In line with standard procedures adopted for eQTL analysis [8, 25, 26], we normalized expression abundance for 10,332 genes using the TMM method [16]. First, we used the function “calcNormFactors” from the R package “edgeR” [27, 32] to calculate the normalization factors then we multiplied the normalization factors by the respective library size. Next, we used the function “cpm” with the normalized library size to obtain TMM normalized counts per million. Next, we carried out an inverse normal transformation [8, 25, 26] using the “RankNorm” function from the R package “RNOmni”. Additive and ANOVA analyses were carried out independently for eQTL analysis with the R package “MatrixEQTL” [51] using 6216 SNPs. In both models, we used genotypes as a fixed effect. We inferred a significant eQTL when the nominal *P*-value was less than 5 × 10^–8^, which is a threshold commonly applied to genome-wide association studies [52–56], and corresponded to a false discovery rate [57] of 4% and 12% for the ANOVA and additive model, respectively.

#### Approach 2: using a differential gene expression framework

We analyzed the RNA-sequencing data with a general linear model in “edgeR” and tested for differential gene expression using the quasi-likelihood F-test [58, 59]. We note that the normalization adopted by default in “edgeR” adjusts for library sequencing depth, but we added the TMM normalization factors calculated by the function “calcNormFactors” to the procedure for identification of eQTLs.

As part of our proposed approach, we also eliminated genes that had outlier values of transcript abundance, which reduced the transcriptome data to 4,149 genes. For these analyses, gene expression data were used as the dependent variable. Genotypes and collection sites were included in the model as independent variables (fixed effects). For additive analysis, the genotypes were input as numerical variables. For ANOVA-like analysis, we carried out a two-tier analysis. First, we tested the association between SNP and gene transcript abundance using all three genotypes as a factor variable. Next, we subset SNPs that were significantly associated with gene transcript abundance and pseudo-coded the genotypes to establish two contrasts [60]. The first contrast compared the homozygote genotype from the reference allele versus the heterozygote and the homozygote genotype from the alternate allele (i.e., AA versus AB, BB). The second contrast compared the homozygote genotype from the alternate allele versus the heterozygote and the homozygote genotype from the reference allele (i.e., AA, AB versus BB). We also inferred a significant eQTL when the nominal *P*-value was less than 5 × 10^–8^ [52–56].

### Visualization of the results

We used the R packages “ggplot2”, “cowplot” [61], or “plotly” [62] for plotting [63] and used Cytoscape [64] to visualize eQTLs in network style.

### Analysis of gene ontology enrichment

We tested several lists of genes for the enrichment of gene ontology using the R package “GOseq”[65]. In order to account for multiple hypothesis testing, *P*-values were adjusted by family wise error rate (FWER) [66]. Results were maintained if they had FWER < 0.05.

## Results

### Overview of SNP identification

We compiled genotype data at 23,506,613 nucleotide positions. Not surprisingly, 99.6% of the genomic positions were homozygous for the reference allele and 2,167 positions were homozygous for the alternate allele. Our pipeline identified 91,006 nucleotide positions showing polymorphisms in our samples. After testing for the deviation of Hardy–Weinberg equilibrium (Fig. 1B), we retained 6,207 SNPs further analysis (Fig. 1C).

Notably, 96% (*n* = 5964) of the SNPs have been previously identified and are recorded in the Ensembl variant database [45, 67], which includes the dbSNP ([68] version 150), while 243 SNPs were not identified in Ensembl variant database (Additional file 2). Most of the SNPs are in 3 prime UTRs (*n* = 1553), and a smaller proportion (*n* = 483) were annotated as missense variants (Additional file 2). We observed no genetic substructure of the individuals based on the SNPs analyzed here (Additional file 3: Fig. S1).

### eQTL analyses

For eQTL analysis, we obtained the matrix with raw counts from a previous study [35] from our group. After filtering for lowly expressed genes, we quantified the transcript abundance for 10,332 protein-coding genes. We then analyzed the transcriptome and the SNP data following the two frameworks.

#### Approach 1: TMM normalized and normal-transformed RNA-seq data

The inverse normal transformation within a gene and across samples [26] indeed normalized the RNA-sequencing data (Additional file 3: Fig S2). Using the R package “MatrixEQTL” [69], the ANOVA and additive analyses concluded in 4.699 and 2.473 s respectively using one core processor (2.60 GHz).

**Fig. 2:**
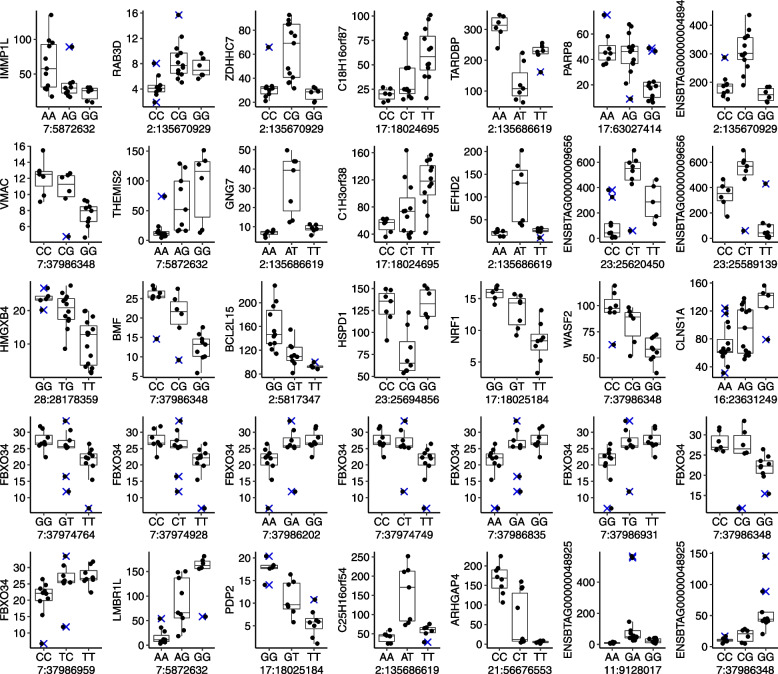
eQTLs identified using ANOVA model on TMM normalized counts per million and normal-transformed RNA-seq data. *Y* axis for all graphs is TMM normalized transcripts per million

**Fig. 3:**
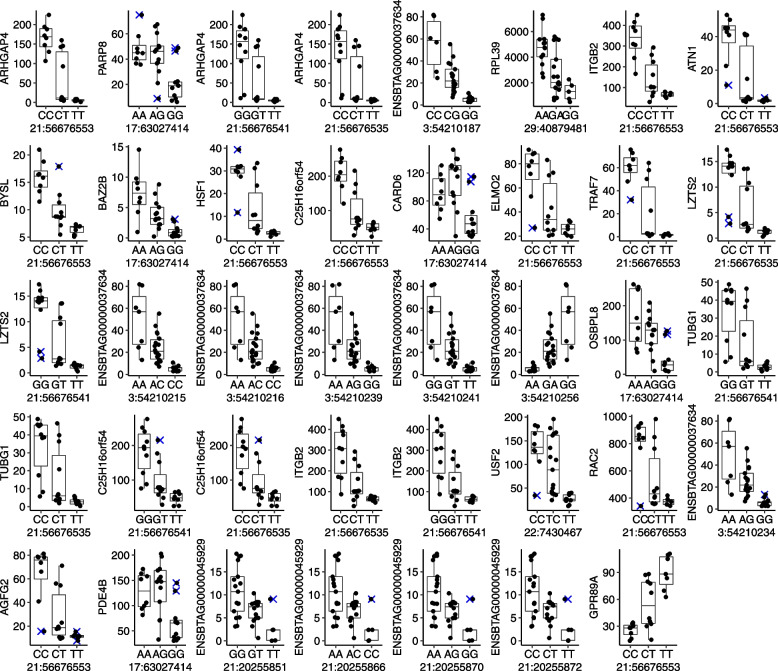
eQTLs identified using additive model on TMM normalized counts per million and normal-transformed RNA-seq data. *Y* axis for all graphs is TMM normalized transcripts per million

We identified 35 significant eQTLs (*P* < 5 × 10^–8^) following the ANOVA model (Fig. 2). Annotated SNPs mapped to the genes: *ASCC1*, *BOLA-DQB*, *FAF2*, *IARS2*, *MGST2*, *MRPS9*, *NECAP2*, *TRIP11* (Additional file 4). We also identified 39 significant eQTLs (*P* < 5 × 10^–8^) following the additive model (Fig. 3). Annotated SNPs mapped to the genes *AHNAK*, *GLB1*, *TRIP11* (Additional file 5), and most of the SNPs on the gene *TRIP11* composed the majority of the eQTLs.

#### Approach 2: using a differential gene expression framework

Using the R package “edgeR” [27], all tests to determine dominance and additive models were completed in 36 and 9 min respectively using 34 core processors (2.60 GHz). We identified 936 significant eQTLs (*P* < 5 × 10^–8^). These eQTLs were formed by 16 SNPs present in the dbSNP and one SNP that is a putatively new variant (Additional file 2) influencing the transcript abundance of 445 genes. The majority (98.6%) of the eQTLs were formed by SNPs on the gene TATA-Box binding protein associated factor 15 (*TAF15*), followed by 6 eQTLs formed by SNPs on the gene SMG6 nonsense-mediated mRNA decay factor (*SMG6*). The other annotated genes with SNPs forming significant eQTLs were *TRIP11*, *PI4KA*, *LMBR1L*, and *ZNF175*. There was no overlap of significant eQTL between both approaches (Additional file 6, Additional file 3: Fig. S3).

**Fig. 4:**
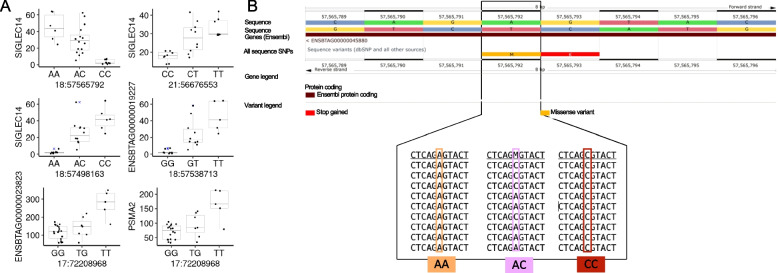
Significant eQTLs were identified using the differential gene expression framework. **A** Network depicting the connectivity between SNPs and the genes whose genotypes are influencing their transcript abundance. **B** Bar plot of the frequency of genes containing SNPs forming eQTLs. Only SNPs that were annotated to genes with a symbol (within a gene model, or within 1,000 nucleotides on each side) are depicted in this figure

It was also possible to separate the eQTLs into dominance or additive allelic interaction. We determined that six of the eQTLs followed the pattern of an additive allelic relationship (Fig. 4A, Additional file 7). Two SNPs (rs41892216 and rs135008768) impacting the expression of the gene sialic acid-binding Ig-like lectin 14 are also present in the region containing the sialic acid-binding Ig-like lectin gene family on chromosome 18. One SNP is a missense mutation (18:57,565,792, Fig. 4B) on the gene *SIGLEC5* and the SNP on nucleotide 18:57,498,163 is a variant downstream to *SIGLEC6*. Two other SNPs were annotated to the genes *PI4KA* (17:72,208,968, rs133672368), *TRIP11* (21:56,676,553, rs479089277) and *ZNF175* (18:57,538,713, rs109161398).

**Fig. 5:**
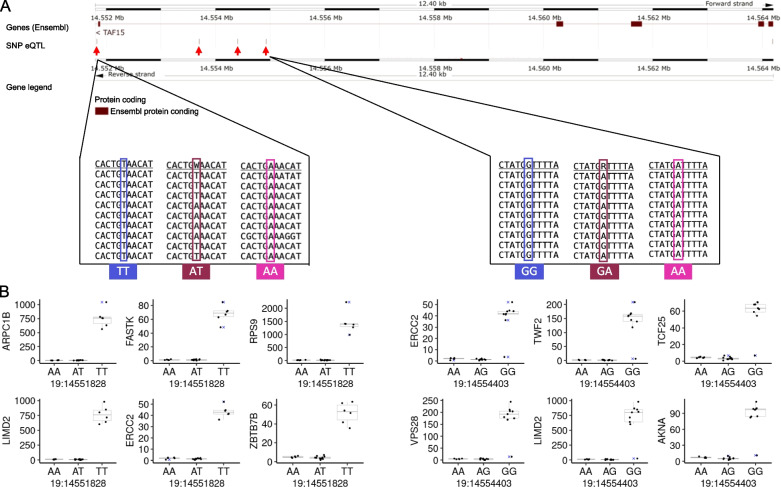
Significant eQTLs were inferred using the differential gene expression framework following the additive relationship between alleles. **A** Eight eQTLs following the additive model determined by edgeR. *Y* axis for all graphs is TMM normalized transcripts per million. **B** Ensembl genome browser indicating the SNP position and examples of raw data used for the SNP’s identification

We also identified 930 significant eQTLs following a dominance allelic relationship (Additional file 8). Eight annotated SNPs mapped to the genes (*LMBR1L*, *SMG6*, *TAF15*, and *TRIP11*). Of notice, four intronic variants on the gene *TAF15* (19:14,551,828, 19:14,554,927, 19:14,554,403, and 19:14,553,701, Fig. 5A) were collectively associated with the expression of 427 genes, with some examples depicted in Fig. 5B.

Given the number of genes expressed in PWBCs that were influenced by SNPs, we asked if there would be an enrichment of gene ontology [70] biological processes among these 427 genes. We observed that by setting a more stringent threshold of significance for the eQTLs (*P* < 5 × 10^–10^), we subset 196 genes, which are enriched for two biological processes (FWER < 0.05: regulation of catalytic activity (fold-enrichment: 3.54; genes: *APBA3*, *ARHGDIA ARHGEF1*, *CAPN1*, *DENND1C*, *EEF1D*, *EIF2B3*, *RAB3IP*, *RALGDS*, *RING1*, Additional file 9), and endocytic recycling (fold-enrichment: 7.81, genes: *CCDC22*, *DENND1C*, *PTPN23*, *SNX12*).

## Discussion

The major goal of our work was to identify genes expressed in PWBCs of crossbred beef heifers whose transcript abundance is impacted by genetic variants. We used a gold standard approach presented by the GTEx consortium, but also analyzed the RNA-sequencing data without a transformation to force a Gaussian distribution of the counts. The framework for eQTL analysis presented here is motivated by the following rationale: (i) the vast majority of eQTL analyses carried out currently use RNA-sequencing data; (ii) by the nature of the procedures, RNA-sequencing data is count data, which is not normally distributed [71, 72]; and (iii) in principle, an eQTL analysis is an expansion of a differential gene expression (DGE) analysis, where samples are grouped by their genotypes, which is analogous to groups or treatments typically used in DGE analysis. Compared to the latest GTEx framework, our analysis of RNA-sequencing data from cattle PWBCs using the DGE framework identified more eQTLs under the dominance model and an equivalent number of eQTLs under the additive model of allele interaction when compared to the framework used in the human or farm GTEx consortia.

Our study has a few limitations, but they do not hinder the validity of our findings. First, we identified SNPs using the RNA-sequencing data, thus we are not accounting for genomic variants in promoters or distal *cis*-regulatory elements. This is likely to have impacted the limited number of *cis*-eQTLs reported here. Second, our transcriptome data represents a mixture of white cells identified in the blood. The proportion of different cells that compose the mixture of white cells was not accounted in our model. A genetic factor contributing to a potential greater abundance of one specific cell type [73] is thus a confounding factor in our study. However, these two limitations do not directly impact our main take home message that there is no need for researchers to normalize RNA-sequencing data in eQTL studies.

### Variant genotyping using RNA-sequencing data

RNA-sequencing data is feasible for the identification of genomic variants in a wide range of organisms, including livestock [74–77], and multiple pipelines have been developed for variant discovery and genotype calling [74–77]. Here we opted for a hybrid approach, which utilized the “SplitNCigarReads” function of GATK followed by the functions “mpileup” and “call” from BCFtools . The reason for using BCFtools was that it calls genotypes at every nucleotide position by default so that individuals were genotyped regardless of the homozygote or heterozygote makeup.

Prior research showed that the efficacy of genotype calling using RNA-sequencing data is high [78]. Although we did not assess the specificity of genotype calling with an orthogonal method, we employed a stringent requirement for coverage equal to or greater than 20×, which is higher than the previously suggested 10× [75, 78] for high confidence genotype calling. In addition, 96% of the variants identified in our pipeline are present in the dbSNP ([68] version 150), and the variants have the same allelic composition reported in the dbSNP. Our hybrid pipeline efficiently genotyped individuals at homozygote and heterozygote genomic positions, although further confirmation is required for the variants called in our work that are not reported in the dbSNP.

### eQTL analysis using RNA-sequencing with and without forcing the data into a Gaussian distribution

Current statistical approaches employed for eQTL analysis [79] assume that the data is normally distributed, and the transformation of RNA-sequencing data to enforce a normal distribution is employed in nearly all major eQTL studies. Our comparison of the RNA-sequencing data prior to and after transforming the data (Additional file 3: Fig. S1) does confirm that the inverse normal transformation [26] is highly effective in reducing skewness and shrinking the variance to reduce the impact of extreme values in the analysis [72], and thus making the data suitable for statistics tests requiring normally distributed data.

We first analyzed our data following the GTEx framework [8], transforming the data to achieve a normal distribution. Our analysis yielded less significant associations between genotype and gene transcript abundance relative to previously published studies that worked with genes expressed in blood samples [80–83] and the recent results from the cattle GTEx consortium[10]. This large difference was expected because we only utilized 6207 SNPs in our analysis, which yields less genotypic data as compared to high-throughput genotyping platforms or imputation of SNPs from reference populations. Another difference between our procedure and other reports was the stringent threshold to infer significance (*P* = 5 × 10^–8^, −log_10_(5 × 10^–8^) = 7.3).

We noted, however, that visual inspection of the data with significant eQTLs identified with the ANOVA model (see examples in Fig. 2C) does not clearly indicate patterns of data distribution that resemble the definition of allelic interaction characterized as complete dominance [84, 85]. The dispersion of the data with significant eQTLs identified with the additive model (see examples in Fig. 3C) does indicate patterns of data distribution that resemble alleles interacting in additive mode [84, 85]. However, the distribution of heterozygotes showed two groups of samples with district profiles.

The graph profiles obtained from significant eQTLs using the GTEx framework prompted us to analyze the data using a DGE framework. To that end, we carried out an analysis using one of the commonly used statistical algorithms coded in the R package “edgeR” [27, 32, 86]. The comparison of our eQTL analysis using “edgeR” showed a striking contrast with the analysis using the GTEx framework and “MatrixEQTL” in many important aspects. First, there was no overlap of significant eQTLs obtained between the two approaches within this study. Here, we point out that identifying which eQTL is true is virtually impossible without further mechanistic experiments that confirm the influence of allelic variants on gene expression [87, 88]. Our findings add to previous observations that the type of statistical analysis carried out is a critical contributor to the lack of replicability observed across eQTL studies [89, 90]. Second, working with specific contrasts, we were able to identify trans eQTLs that more closely resemble complete dominance, which were not identified by the standard framework. Our results are evidence that the number of genes whose expression are under genetic control and follow patterns of complete dominance [91, 92] is probably more common than previously expected [8]. The identification of groups of genes enriched for specific biological processes strongly supports that this genetic control under the dominance model may have a biological role in the function of PWBCs.

We identified two important aspects that show a contrast between the ANOVA framework and the DGE framework we propose here. First, the functions in "MatrixEQTL” require less computational resources and time to conclude the analysis relative to the calculations carried out using the DGE framework in “edgeR”. Our proposed approach is inherently more complex, as we carried out multiple tests to provide robust and valuable information about dominance interaction between alleles. It is also very important to note that our study is not about the tools (“MatrixEQTL” or “edgeR”), because researchers can use other tools for the standard analysis of eQTL such as “FastQTL” [93] or DESeq2 [20] for the DGE framework. Second, the transformation of the data to fit a normal distribution clearly shrinks the variance (Additional file 3, Fig. S4), reducing the differences in transcript abundance among genotypes thus reducing the likelihood of these eQTLs to be inferred as significant. In the end, the most critical choice researchers need to make is between (i) forcing data that is not normally distributed and has many outlier data points [71, 72] into normality or (ii) utilizing a framework that employs a statistical test appropriate for count data.

## Conclusions

In summary, different types of data normalization and analytical procedures lead to a variety of combinations that can be used for eQTL analysis using RNA-sequencing. Most of these approaches also transform the data to fit a normal distribution. Our analysis showed that it is possible to carry out eQTL studies using the concepts and analytical framework developed for differential gene expression that does not require data transformation to fit a normal distribution, thus it is likely more suitable for RNA-sequencing. The approach proposed here can uncover genetic control of gene expression that is biologically relevant for the tissue studied that otherwise may not be detected through data transformation and linear models.

## Supplementary Information

**Additional file 1.** Supplementary code to Robust identification of regulatory variants (eQTLs) using a differential expression framework developed for RNA-sequencing. All codes utilized for in our work to produce the results described in the paper.

**Additional file 2.** Distribution of the SNPs based on presence in the database and predicted consequence of the variant.

**Additional file 3: Fig. S1.** Principal component analysis of the samples based on the SNP data. **Fig. S2.** Representation of RNA-Sequencing data after (A) normalization of the count data with the TMM method and adjustment per million reads, and normalization as demonstrated by the (B) histogram and (C) qqplot. **Fig. S3.** Scatterplot of the of the raw *P* values (-Log10() transformed) for the eQTLs following the (A) ANOVA model, and (B) additive model. **Fig. S4.** Plots of significant eQTLs following the dominance mode of allelic interaction identified by the DGE framework. (A) Raw counts (B) Transcript per million (C) TMM normalized counts per million. (D) TMM normalized counts per million and normal transformed.

**Additional file 4.** Annotated results of eQTL analysis using the GTEx framework and ANOVA model.

**Additional file 5.** Annotated results of eQTL analysis using the GTEx framework and additive model.

**Additional file 6.** eQTL results with DGE framework and the standard approach using MatrixEQTL.

**Additional file 7.** Annotated eQTL results using edgeR framework and additive model.

**Additional file 8.** Annotated eQTL results with edgeR framework and ANOVA model.

**Additional file 9.** Gene ontology analysis of genes whose expression are influenced by SNPs.

## Data Availability

The datasets analyzed during the current study are available in the GEO repository, session identifiers: GSE103628 and GSE146041.

## Abbreviations

ANOVA: Analysis of variance
CPM: Counts per million reads
DGE: Differential gene expression
eQTL: Expression quantitative trait loci
FPKM: Fragments per kilobase per million reads
FWER: Family wise error rate
GTEx: Genotype-Tissue Expression
log_2_: Logarithm base 2
n: Number
P: Probability
PWBCs: Peripheral white blood cells
RNA: Ribonucleic acid
SNP: Single nucleotide polymorphism
TMM: Trimmed means of M-values
TPM: Transcript per million reads
UTRs: Untraslated regions

## References

[1] Shi H, Kichaev G, Pasaniuc B (2016). Contrasting the genetic architecture of 30 complex traits from summary association data. Am J Hum Genet.

[2] Shi H, Burch KS, Johnson R, Freund MK, Kichaev G, Mancuso N (2020). Localizing components of shared transethnic genetic architecture of complex traits from GWAS summary data. Am J Hum Genet.

[3] Goddard ME, Kemper KE, MacLeod IM, Chamberlain AJ, Hayes BJ (2016). Genetics of complex traits: prediction of phenotype, identification of causal polymorphisms and genetic architecture. Proc Biol Sci.

[4] Eyre-Walker A (2010). Evolution in health and medicine Sackler colloquium: Genetic architecture of a complex trait and its implications for fitness and genome-wide association studies. P Natl Acad Sci USA.

[5] Watanabe K, Stringer S, Frei O, UmicevicMirkov M, de Leeuw C, Polderman TJC (2019). A global overview of pleiotropy and genetic architecture in complex traits. Nat Genet.

[6] Williams RB, Chan EK, Cowley MJ, Little PF (2007). The influence of genetic variation on gene expression. Genome Res.

[7] Kreitmaier P, Katsoula G, Zeggini E (2023). Insights from multi-omics integration in complex disease primary tissues. Trends Genet.

[8] Consortium GT (2020). The GTEx Consortium atlas of genetic regulatory effects across human tissues. Science.

[9] Kim-Hellmuth S, Aguet F, Oliva M, Munoz-Aguirre M, Kasela S, Wucher V (2020). Cell type-specific genetic regulation of gene expression across human tissues. Science..

[10] Liu S, Gao Y, Canela-Xandri O, Wang S, Yu Y, Cai W (2022). A multi-tissue atlas of regulatory variants in cattle. Nat Genet.

[11] The GTEx Consortium, Ardlie KG, Deluca DS, Segrè AV, Sullivan TJ, Young TR. The Genotype-Tissue Expression (GTEx) pilot analysis: multitissue gene regulation in humans. Science. 2015;348:648–60. 10.1126/science.1262110 PMC4547484 25954001

[12] Gregersen PK (2009). Closing the gap between genotype and phenotype. Nat Genet.

[13] Dendrou CA, Plagnol V, Fung E, Yang JH, Downes K, Cooper JD (2009). Cell-specific protein phenotypes for the autoimmune locus IL2RA using a genotype-selectable human bioresource. Nat Genet.

[14] Nica AC, Dermitzakis ET (2013). Expression quantitative trait loci: present and future. Philos Trans R Soc Lond B Biol Sci.

[15] Kendziorski CM, Chen M, Yuan M, Lan H, Attie AD (2006). Statistical methods for expression quantitative trait loci (eQTL) mapping. Biometrics.

[16] Robinson MD, Oshlack A (2010). A scaling normalization method for differential expression analysis of RNA-seq data. Genome Biol.

[17] Mortazavi A, Williams BA, McCue K, Schaeffer L, Wold B (2008). Mapping and quantifying mammalian transcriptomes by RNA-Seq. Nat Methods.

[18] Li B, Dewey CN (2011). RSEM: accurate transcript quantification from RNA-Seq data with or without a reference genome. BMC Bioinformatics.

[19] Yang J, Wang D, Yang Y, Yang W, Jin W, Niu X (2021). A systematic comparison of normalization methods for eQTL analysis. Brief Bioinform..

[20] Love MI, Huber W, Anders S (2014). Moderated estimation of fold change and dispersion for RNA-seq data with DESeq2. Genome Biol.

[21] Mason VC, Schaefer RJ, McCue ME, Leeb T, Gerber V (2018). eQTL discovery and their association with severe equine asthma in european warmblood horses. BMC Genomics.

[22] Zeng B, Lloyd-Jones LR, Montgomery GW, Metspalu A, Esko T, Franke L (2019). Comprehensive multiple eQTL detection and its application to GWAS interpretation. Genetics.

[23] Strunz T, Grassmann F, Gayan J, Nahkuri S, Souza-Costa D, Maugeais C (2018). A mega-analysis of expression quantitative trait loci (eQTL) provides insight into the regulatory architecture of gene expression variation in liver. Sci Rep.

[24] Albert FW, Bloom JS, Siegel J, Day L, Kruglyak L (2018). Genetics of trans-regulatory variation in gene expression. Elife..

[25] Kerimov N, Hayhurst JD, Peikova K, Manning JR, Walter P, Kolberg L (2021). A compendium of uniformly processed human gene expression and splicing quantitative trait loci. Nat Genet.

[26] Beasley TM, Erickson S, Allison DB (2009). Rank-based inverse normal transformations are increasingly used, but are they merited?. Behav Genet.

[27] McCarthy DJ, Smyth GK (2010). edgeR: a Bioconductor package for differential expression analysis of digital gene expression data. Bioinformatics.

[28] Hardcastle TJ, Kelly KA (2010). baySeq: empirical bayesian methods for identifying differential expression in sequence count data. BMC Bioinformatics.

[29] Robinson MD, Smyth GK (2007). Moderated statistical tests for assessing differences in tag abundance. Bioinformatics.

[30] Anders S, Huber W (2010). Differential expression analysis for sequence count data. Genome Biol.

[31] Gentleman RC, Carey VJ, Bates DM, Bolstad B, Dettling M, Dudoit S (2004). Bioconductor: open software development for computational biology and bioinformatics. Genome Biol.

[32] McCarthy DJ, Chen Y, Smyth GK (2012). Differential expression analysis of multifactor RNA-Seq experiments with respect to biological variation. Nucleic Acids Res.

[33] Dickinson SE, Biase FH (2018). Transcriptome data of peripheral white blood cells from beef heifers collected at the time of artificial insemination. Data Brief.

[34] Dickinson SE, Griffin BA, Elmore MF, Kriese-Anderson L, Elmore JB, Dyce PW (2018). Transcriptome profiles in peripheral white blood cells at the time of artificial insemination discriminate beef heifers with different fertility potential. BMC Genomics.

[35] Moorey SE, Walker BN, Elmore MF, Elmore JB, Rodning SP, Biase FH (2020). Rewiring of gene expression in circulating white blood cells is associated with pregnancy outcome in heifers (Bos taurus). Sci Rep.

[36] Bolger AM, Lohse M, Usadel B (2014). Trimmomatic: a flexible trimmer for Illumina sequence data. Bioinformatics.

[37] Kim D, Paggi JM, Park C, Bennett C, Salzberg SL (2019). Graph-based genome alignment and genotyping with HISAT2 and HISAT-genotype. Nat Biotechnol..

[38] Elsik CG, Tellam RL, Worley KC, Gibbs RA, Muzny DM, Weinstock GM (2009). The genome sequence of taurine cattle: a window to ruminant biology and evolution. Science.

[39] Rosen BD, Bickhart DM, Schnabel RD, Koren S, Elsik CG, Tseng E (2020). De novo assembly of the cattle reference genome with single-molecule sequencing. GigaScience..

[40] Flicek P, Amode MR, Barrell D, Beal K, Billis K, Brent S (2014). Ensembl.

[41] Li H (2011). A statistical framework for SNP calling, mutation discovery, association mapping and population genetical parameter estimatin from sequencing data. Bioinformatics.

[42] Tischler G, Leonard S (2014). biobambam: tools for read pair collation based algorithms on BAM files. Source Code Biol Med..

[43] Auwera GAVd, O'Connor BD. Genomics in the cloud: using Docker, GATK, and WDL in Terra. 1st ed. O'Reilly Media; 2020.

[44] Ihaka R, Gentleman R (1996). R: a language for data analysis and graphics. J Comput Graph Stat.

[45] McLaren W, Gil L, Hunt SE, Riat HS, Ritchie GR, Thormann A (2016). The ensembl variant effect predictor. Genome Biol.

[46] Wagner GP, Kin K, Lynch VJ (2012). Measurement of mRNA abundance using RNA-seq data: RPKM measure is inconsistent among samples. Theory Biosci.

[47] Purcell S, Neale B, Todd-Brown K, Thomas L, Ferreira MA, Bender D (2007). PLINK: a tool set for whole-genome association and population-based linkage analyses. Am J Hum Genet.

[48] Price AL, Patterson NJ, Plenge RM, Weinblatt ME, Shadick NA, Reich D (2006). Principal components analysis corrects for stratification in genome-wide association studies. Nat Genet.

[49] Huang QQ, Ritchie SC, Brozynska M, Inouye M. Power, false discovery rate and winner’s curse in eQTL studies. Nucleic Acids Res. 2018;46:e133. 10.1093/nar/gky780 PMC6294523 30189032

[50] Graffelman J (2015). Exploring diallelic genetic markers: The HardyWeinberg package. J Stat Softw.

[51] Shabalin AA (2012). Matrix eQTL: Ultra fast eQTL analysis via large matrix operations. Bioinformatics..

[52] Wood AR, Esko T, Yang J, Vedantam S, Pers TH, Gustafsson S (2014). Defining the role of common variation in the genomic and biological architecture of adult human height. Nat Genet.

[53] Vicente CT, Revez JA, Ferreira MAR (2017). Lessons from ten years of genome-wide association studies of asthma. Clin Transl Immunol.

[54] Dudbridge F, Gusnanto A (2008). Estimation of significance thresholds for genome-wide association scans. Genet Epidemiol.

[55] Pe’er I, Yelensky R, Altshuler D, Daly MJ. Estimation of the multiple testing burden for genome-wide association studies of nearly all common variants. Genet Epidemiol. 2008;32:381–5. 10.1002/gepi.20303 18348202

[56] Altshuler D, Brooks LD, Chakravarti A, Collins FS, Daly MJ, Donnelly P (2005). A haplotype map of the human genome. Nature.

[57] Benjamini Y, Hochberg Y (1995). Controlling the false discovery rate - a practical and powerful approach to multiple testing. J Roy Stat Soc B Met.

[58] Lund SP, Nettleton D, McCarthy DJ, Smyth GK. Detecting differential expression in RNA-sequence data using quasi-likelihood with shrunken dispersion estimates. Stat Appl Genet Mol Biol. 2012;11(5). 10.1515/1544-6115.1826. 10.1515/1544-6115.1826 23104842

[59] Lun ATL, Chen YS, Smyth GK. It’s DE-licious: a recipe for differential expression analyses of RNA-seq experiments using quasi-likelihood methods in edgeR. Methods Mol Biol. 2016;1418:391–416. 10.1007/978-1-4939-3578-9_19 27008025

[60] Horita N, Kaneko T (2015). Genetic model selection for a case-control study and a meta-analysis. Meta Gene.

[61] cowplot: Streamlined plot theme and plot Annotations for ggplot2. https://wilkelab.org/cowplot/

[62] Sievert C. Interactive web-based data visualization with R, plotly, and shiny. 1st Edition. New York: Chapman and Hall/CRC; 2020. 10.1201/9780429447273.

[63] Wickham H (2009). ggplot2: elegant graphics for data analysis.

[64] Shannon P, Markiel A, Ozier O, Baliga NS, Wang JT, Ramage D (2003). Cytoscape: a software environment for integrated models of biomolecular interaction networks. Genome Res.

[65] Young MD, Wakefield MJ, Smyth GK, Oshlack A (2010). Gene ontology analysis for RNA-seq: accounting for selection bias. Genome Biol..

[66] Holm S (1979). A simple sequentially rejective multiple test procedure. Scand Stat Theory Appl.

[67] Hunt SE, Moore B, Amode RM, Armean IM, Lemos D, Mushtaq A, et al. Annotating and prioritizing genomic variants using the ensembl variant effect predictor-a tutorial. Hum Mutat. 2021;43:986–97. 10.1002/humu.24298 PMC7613081 34816521

[68] Sherry ST, Ward M, Sirotkin K (1999). dbSNP-database for single nucleotide polymorphisms and other classes of minor genetic variation. Genome Res.

[69] Shabalin AA (2012). Matrix eQTL: ultra fast eQTL analysis via large matrix operations. Bioinformatics.

[70] Ashburner M, Ball CA, Blake JA, Botstein D, Butler H, Cherry JM (2000). Gene ontology: tool for the unification of biology. Gene Ontol Consortium Nat Genet.

[71] Noel-MacDonnell JR, Usset J, Goode EL, Fridley BL (2018). Assessment of data transformations for model-based clustering of RNA-Seq data. PLoS ONE.

[72] Zwiener I, Frisch B, Binder H (2014). Transforming RNA-Seq data to improve the performance of prognostic gene signatures. PLoS ONE.

[73] Jain D, Hodonsky CJ, Schick UM, Morrison JV, Minnerath S, Brown L (2017). Genome-wide association of white blood cell counts in Hispanic/Latino Americans: the Hispanic community health study/study of Latinos. Hum Mol Genet.

[74] Jehl F, Degalez F, Bernard M, Lecerf F, Lagoutte L, Desert C (2021). RNA-Seq data for reliable SNP detection and genotype calling: interest for coding variant characterization and cis-regulation analysis by allele-specific expression in livestock species. Front Genet.

[75] Lam S, Zeidan J, Miglior F, Suarez-Vega A, Gomez-Redondo I, Fonseca PAS (2020). Development and comparison of RNA-sequencing pipelines for more accurate SNP identification: practical example of functional SNP detection associated with feed efficiency in Nellore beef cattle. BMC Genomics.

[76] Lam S, Miglior F, Fonseca PAS, Gomez-Redondo I, Zeidan J, Suarez-Vega A (2021). Identification of functional candidate variants and genes for feed efficiency in Holstein and Jersey cattle breeds using RNA-sequencing. J Dairy Sci.

[77] Bakhtiarizadeh MR, Alamouti AA (2020). RNA-Seq based genetic variant discovery provides new insights into controlling fat deposition in the tail of sheep. Sci Rep.

[78] Brouard JS, Schenkel F, Marete A, Bissonnette N (2019). The GATK joint genotyping workflow is appropriate for calling variants in RNA-seq experiments. J Anim Sci Biotechnol.

[79] Nodzak C (2020). Introductory methods for eQTL analyses. Methods Mol Biol.

[80] van den Berg I, Hayes BJ, Chamberlain AJ, Goddard ME (2019). Overlap between eQTL and QTL associated with production traits and fertility in dairy cattle. BMC Genomics.

[81] Lee YL, Takeda H, Costa Monteiro Moreira G, Karim L, Mullaart E, Coppieters W (2021). A 12 kb multi-allelic copy number variation encompassing a GC gene enhancer is associated with mastitis resistance in dairy cattle. Plos Genet..

[82] Fang L, Cai W, Liu S, Canela-Xandri O, Gao Y, Jiang J (2020). Comprehensive analyses of 723 transcriptomes enhance genetic and biological interpretations for complex traits in cattle. Genome Res.

[83] Canive M, Fernandez-Jimenez N, Casais R, Vazquez P, Lavin JL, Bilbao JR (2021). Identification of loci associated with susceptibility to bovine paratuberculosis and with the dysregulation of the MECOM, eEF1A2, and U1 spliceosomal RNA expression. Sci Rep.

[84] Gjuvsland AB, Plahte E, Adnoy T, Omholt SW (2010). Allele interaction–single locus genetics meets regulatory biology. PLoS ONE.

[85] Elston RC, Satagopan JM, Sun S (2012). Genetic terminology. Methods Mol Biol.

[86] Anders S, McCarthy DJ, Chen Y, Okoniewski M, Smyth GK, Huber W (2013). Count-based differential expression analysis of RNA sequencing data using R and Bioconductor. Nat Protoc.

[87] Hanson C, Cairns J, Wang L, Sinha S (2018). Principled multi-omic analysis reveals gene regulatory mechanisms of phenotype variation. Genome Res.

[88] Doss S, Schadt EE, Drake TA, Lusis AJ (2005). Cis-acting expression quantitative trait loci in mice. Genome Res.

[89] Loguercio S, Overall RW, Michaelson JJ, Wiltshire T, Pletcher MT, Miller BH (2010). Integrative analysis of low- and high-resolution eQTL. PLoS ONE.

[90] Goring HH, Curran JE, Johnson MP, Dyer TD, Charlesworth J, Cole SA (2007). Discovery of expression QTLs using large-scale transcriptional profiling in human lymphocytes. Nat Genet.

[91] Wilkie AO (1994). The molecular basis of genetic dominance. J Med Genet.

[92] Kacser H, Burns JA (1981). The molecular basis of dominance. Genetics.

[93] Ongen H, Buil A, Brown AA, Dermitzakis ET, Delaneau O (2016). Fast and efficient QTL mapper for thousands of molecular phenotypes. Bioinformatics.

