# Supplementary material for: Robust identification of regulatory variants (eQTLs) using a differential expression framework developed for RNA-sequencing
Source: J Anim Sci Biotechnol. 2023 May 5;14:62. doi: 10.1186/s40104-023-00861-0 (PMC10161580; doi:10.1186/s40104-023-00861-0)
Supplement: Supplementary file 3 — Additional file 3: Fig. S1. Principal component analysis of the samples based on the SNP data. Fig. S2. Representation of RNA-Sequencing data after (A) normalization of the count data with the TMM method and adjustment per million reads, and normalization as demonstrated by the (B) histogram and (C) qqplot. Fig. S3. Scatterplot of the of the raw P values (-Log10() transformed) for the eQTLs following the (A) ANOVA model, and (B) additive model. Fig. S4. Plots of significant eQTLs following the dominance mode of allelic interaction identified by the DGE framework. (A) Raw counts (B) Transcript per million (C) TMM normalized counts per million. (D) TMM normalized counts per million and normal transformed. [file 40104_2023_861_MOESM3_ESM.pdf]

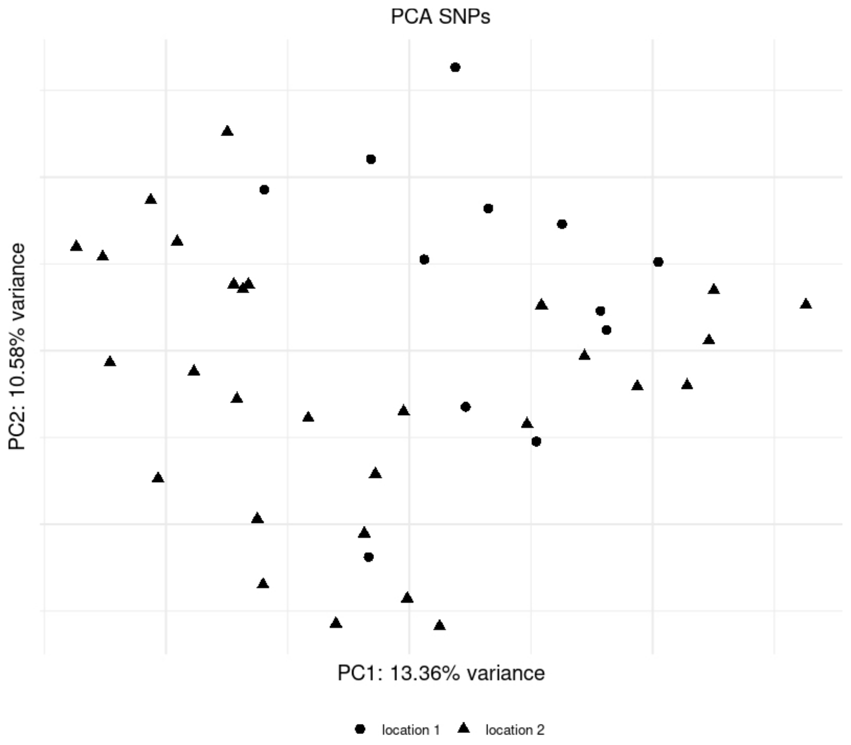

Fig. S1. Principal component analysis of the samples based on the SNP data.

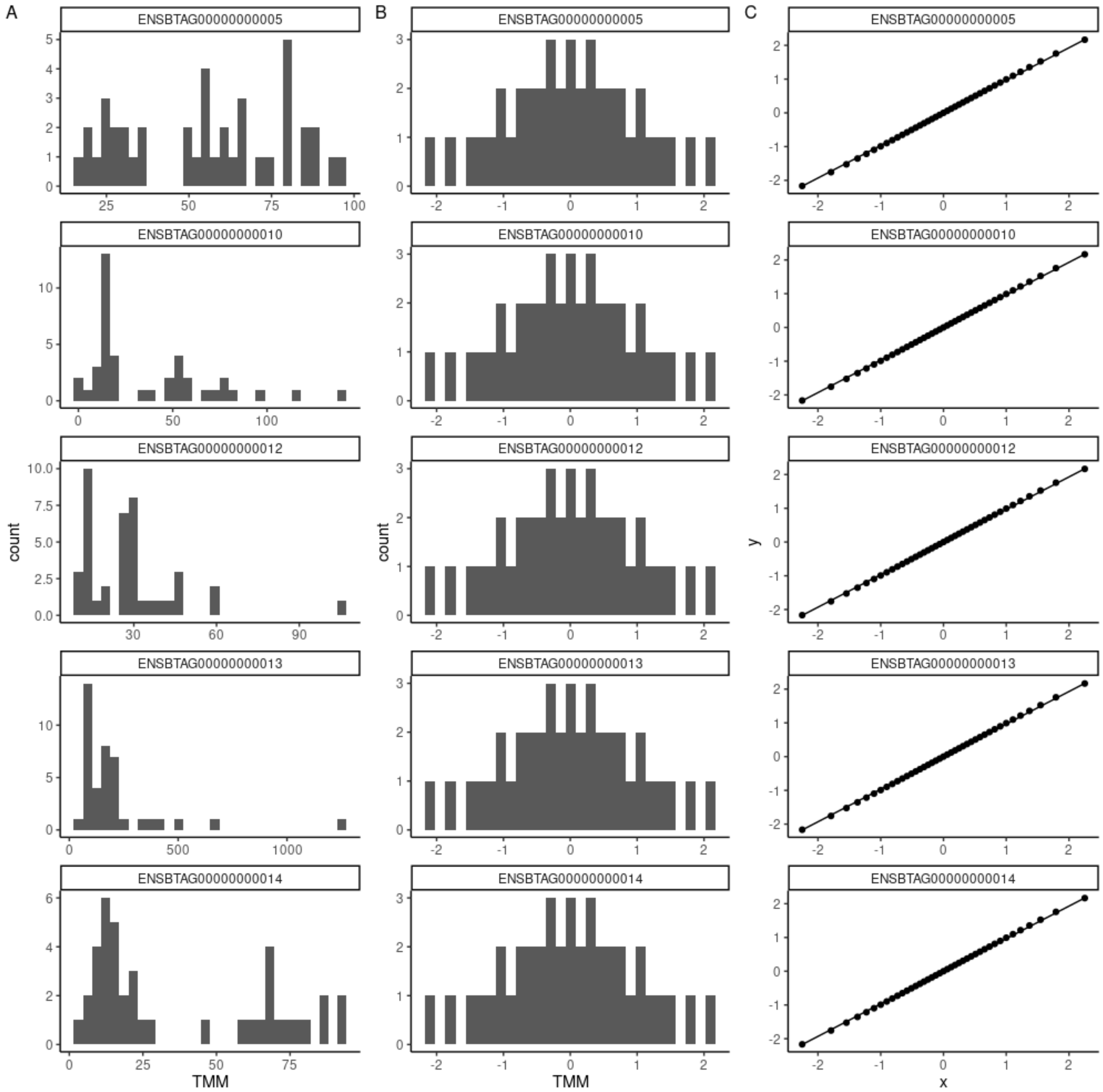

Fig. S2. Representation of RNA-Sequencing data after (A) normalization of the count data with the TMM method and adjustment per million reads, and normalization as demonstrated by the (B) histogram and (C) qqplot.

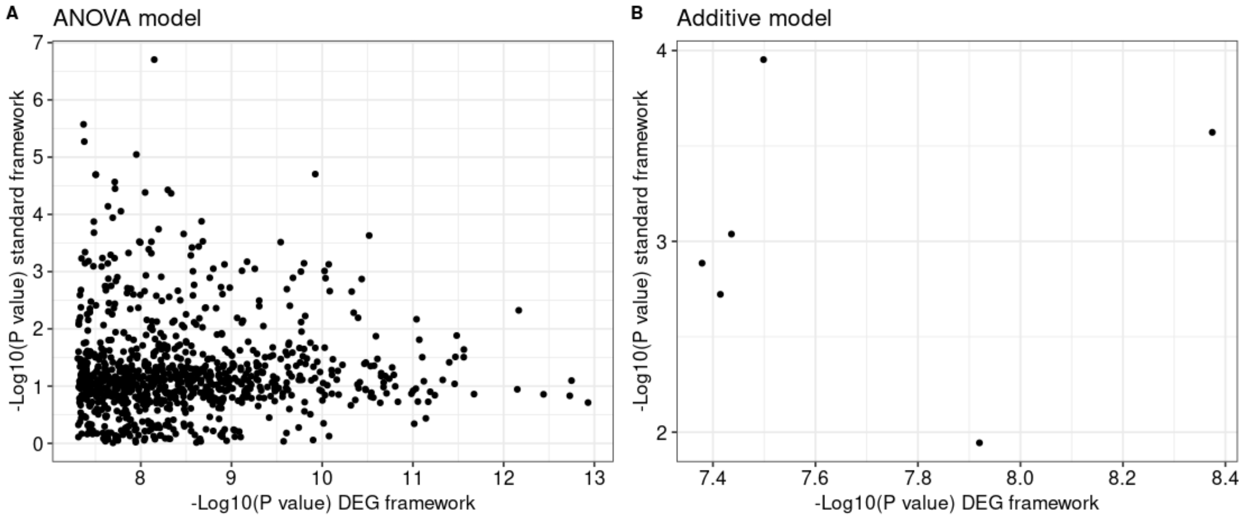

Fig S3. Scatterplot of the of the raw P values (-Log_{10}() transformed) for the eQTLs following the (A) ANOVA model, and (B) additive model.

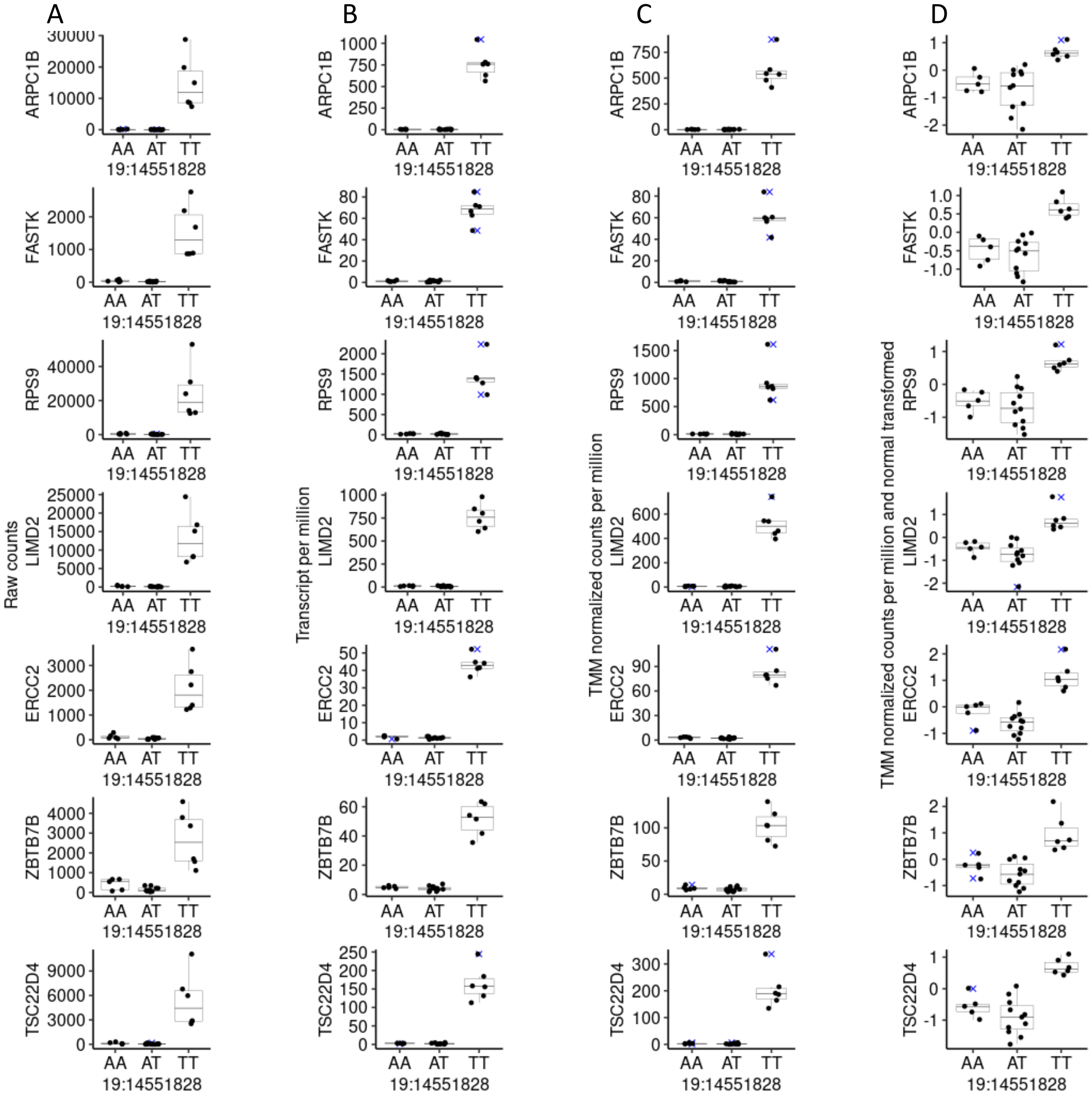

Fig S4. Plots of significant eQTLs following the dominance mode of allelic interaction identified by the DGE framework. (A) Raw counts (B) Transcript per million (C) TMM normalized counts per million. (D) TMM normalized counts per million and normal transformed.
